# Mechanisms of MEHP Inhibitory Action and Analysis of Potential Replacement Plasticizers on Leydig Cell Steroidogenesis

**DOI:** 10.3390/ijms222111456

**Published:** 2021-10-24

**Authors:** Annick N. Enangue Njembele, Jacques J. Tremblay

**Affiliations:** 1 Reproduction, Mother and Child Health, Room T3-67, Centre de Recherche du CHU de Québec–Université Laval CHUL 2705 Laurier Blvd., Québec City, QC G1V 4G2, Canada; enangue7@yahoo.fr; 2 Centre for Research in Reproduction, Development and Intergenerational Health, Department of Obstetrics, Gynecology, and Reproduction, Faculty of Medicine, Université Laval, Québec City, QC G1V 0A6, Canada

**Keywords:** testis, Leydig cells, steroidogenesis, Star, environmental toxicology, endocrine disrupters, phthalates, DEHP, green plasticizers

## Abstract

Steroid production in Leydig cells is stimulated mainly by the pituitary luteinizing hormone, which leads to increased expression of genes involved in steroidogenesis, including the gene encoding the steroidogenic acute regulatory (STAR) protein. Mono(2-ethylhexyl)phthalate (MEHP), the active metabolite of the widely used plasticizer DEHP, is known to disrupt Leydig steroidogenesis but its mechanisms of action remain poorly understood. We found that MEHP caused a significant reduction in hormone-induced steroid hormone production in two Leydig cell lines, MA-10 and MLTC-1. Consistent with disrupted cholesterol transport, we found that MEHP represses cAMP-induced *Star* promoter activity. MEHP responsiveness was mapped to the proximal *Star* promoter, which contains multiple binding sites for several transcription factors. In addition to STAR, we found that MEHP also reduced the levels of ferredoxin reductase, a protein essential for electron transport during steroidogenesis. Finally, we tested new plasticizers as alternatives to phthalates. Two plasticizers, dioctyl succinate and 1,6-hexanediol dibenzoate, had no significant effect on hormone-induced steroidogenesis. Our current findings reveal that MEHP represses steroidogenesis by affecting cholesterol transport and its conversion into pregnenolone. We also found that two novel molecules with desirable plasticizer properties have no impact on Leydig cell steroidogenesis and could be suitable phthalate replacements.

## 1. Introduction

Phthalates or phthalate esters are a family of lipophilic chemicals that are primarily used as plasticizers and additives to improve flexibility and durability of a product. Phthalates are widely used in the manufacture of polyvinyl chloride (PVC) and consequently can be found in several consumer and industrial products [1,2] such as medical devices (blood/fluid bags, intravenous tubing), food wrapping and packaging [3,4], cosmetics [5] (shampoo, perfume) [6], toys [7,8], paint and finishes, and upholstery [9]. It is known that phthalates are not covalently bound to their matrix in PVC and thus are released into the environment (reviewed in [10]). Consequently, phthalates contaminate food and water chains and can be found in human and animal fluids (reviewed in [11,12]). Di(2-ethylhexyl)phthalate (DEHP) is the most abundant phthalate. Approximately 95% of DEHP produced is used as a plasticizer in PVC [13]. In 2004, the worldwide production of DEHP was estimated to be about 2 million metric tons (4.4 billion pounds) (reviewed in [14,15,16]). Despite the controversy surrounding its effects, DEHP consumption continued to rise to an estimated 3.07 million metric tons (6.754 billion pounds) in 2017 (reviewed in [12]).

DEHP is absorbed in the body via three main routes: ingestion, inhalation, and dermal absorption (reviewed in [17]). Although variable, daily DEHP exposure is estimated in humans at 8 µg/kg of body weight in adults and 25 µg/kg of body weight in children (reviewed in [1,18,19,20,21,22]). Inside the body, phthalates—including DEHP—are metabolized by hydrolysis and conjugation and then eliminated in urine (reviewed in [12]). During hydrolysis, DEHP is mainly metabolized like other phthalates by intestinal esterase and lipase into two main components: monoethylhexyl phthalate (MEHP) and ethylhexanol [22,23]. Following oxidation and hydroxylation, MEHP, the most abundant metabolite of DEHP, is further metabolized into other secondary metabolites. Some of these secondary metabolites are eliminated directly in urine, while others are conjugated, mostly by glucuronidation, then eliminated in urine and feces [23]. Some of the conjugated metabolites of DEHP remain in blood plasma [24].

Several studies performed in rodents have revealed that exposure to DEHP/MEHP causes reproductive abnormalities in adult males and in fetuses exposed in utero or during lactation ([25,26,27,28], reviewed in [29]). In rats, in utero exposure to DEHP causes reduced anogenital distance, cryptorchidism, hypospadias, multinucleated germ cells, increased aggregation of Leydig cells, and decreased testosterone production in male offspring [30,31,32,33,34,35]. In humans, exposure of human fetal testis explants in culture to DEHP results in decreased testosterone production [36] and is associated with reduced Leydig cell function [37,38]. In all studies, DEHP toxicity was found to be conferred mainly by MEHP [20,39,40]. Exposure to MEHP was found to decrease testosterone production by targeting Leydig cells [35,40,41,42].

Steroid synthesis in Leydig cells involves a well-characterized biosynthetic pathway requiring the sequential action of multiple enzymes (reviewed in [43]). The initial and rate limiting step in steroidogenesis, stimulated by the steroidogenic acute regulatory protein (STAR), involves cholesterol transport from the outer to the inner mitochondrial membrane where it can be converted to pregnenolone by the enzyme P450 side-chain cleavage (P450SCC, CYP11A1). Pregnenolone is then exported from the mitochondria and imported into the endoplasmic reticulum where it is transformed into testosterone through the sequential action of P450 17α-hydroxylase (CYP17A1), 3β-hydroxysteroid dehydrogenase (HSD3B1), and 17β-hydroxysteroid dehydrogenase type 3 (HSD17B3). Additional proteins are also required to ensure proper functioning of the various enzymes; these include cytochrome b5 (CYT B5), P450 oxidoreductase (POR), ferredoxin (FDX1), and ferredoxin reductase (FDXR, also known as adrenodoxin reductase, ADXR) (reviewed in [43]).

Leydig cell steroidogenesis is stimulated by the binding of the pituitary luteinizing hormone (LH) to its receptor, leading to a rise in cAMP production and activation of multiple signaling pathways and kinases including PKA, ERK, and CAMKI ([44], reviewed in [45,46]). These kinases then phosphorylate transcription factors which upregulate the expression of several genes involved in steroidogenesis (reviewed in [45,46]), including the *Star* gene which codes for the STAR protein (reviewed in [47,48,49,50]). Several studies performed using animal models, rodent testis in culture, and Leydig cell lines revealed that MEHP negatively affects steroidogenesis by decreasing the expression of several genes involved in androgen production, including the *Star* gene [40,41,42,51,52]. The molecular mechanisms of MEHP inhibitory action on *Star* gene expression remain to be fully elucidated. In addition, growing concerns with respect to environmental and health problems caused by phthalates emphasized the necessity to develop greener plasticizers that would be less likely to leach, more quickly degraded, and less toxic as alternatives to phthalates. Although some replacement plasticizers have been developed and used, data regarding their effects are limited (reviewed in [53]).

In the present work, we used two Leydig cell lines, MA-10 and MLTC-1, to study the mechanisms of MEHP action on Leydig cell steroidogenesis. We provide evidence that MEHP targets at least two critical steps of steroidogenesis, cholesterol transport, and its conversion into pregnenolone. We also tested the effects of a series of novel plasticizers as phthalate replacements and found that two had no significant impact on Leydig cell steroidogenesis.

## 2. Results

### 2.1. MEHP Represses Steroidogenesis by Affecting Star Gene Transcription

**Figure 1:**
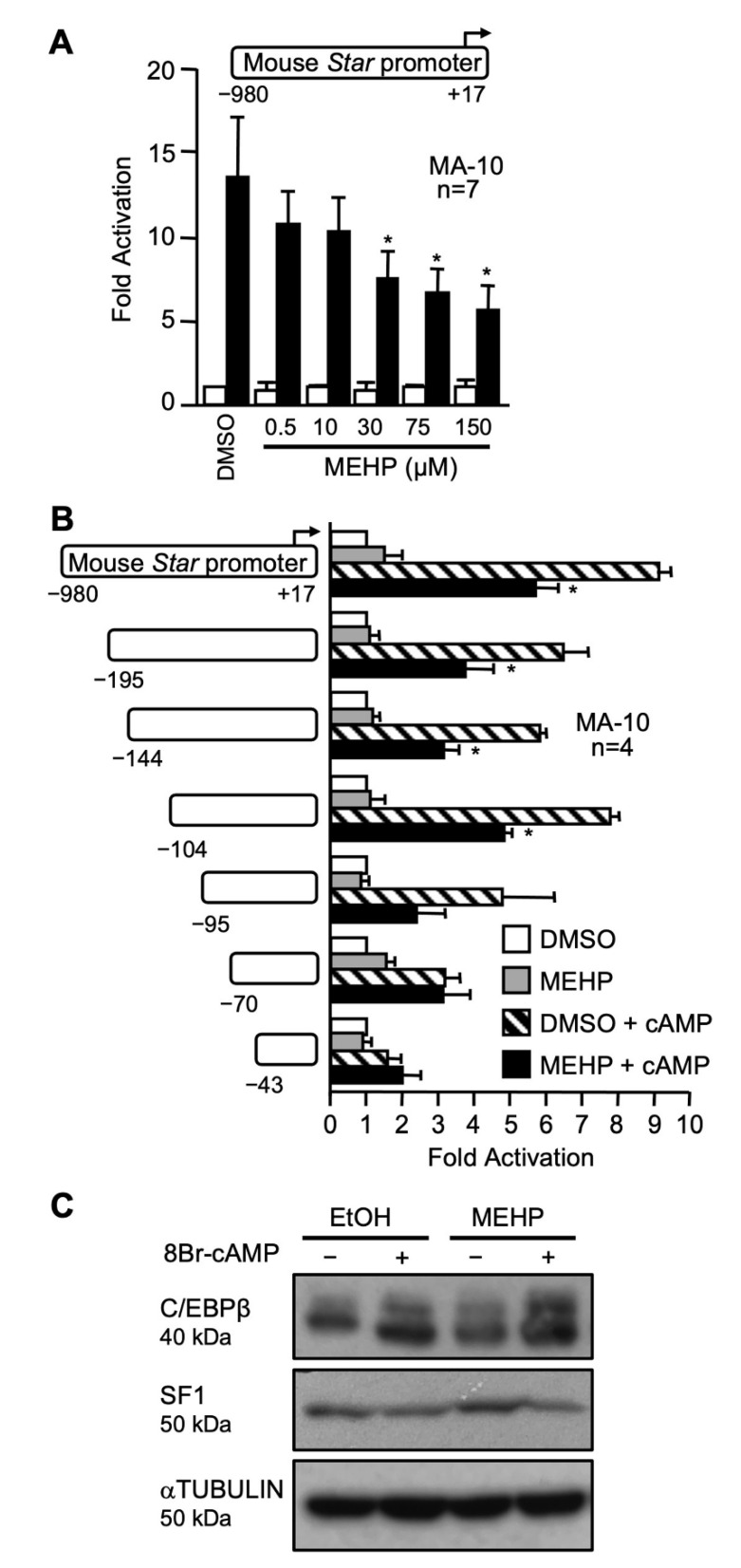
MEHP targets the proximal *Star* promoter in MA-10 Leydig cells. (**A**) MA-10 were transfected with a −980 bp mouse *Star* reporter and treated for 24 h with either dimethylsulfoxide (DMSO) or increasing concentrations of MEHP in the presence (black bars) or absence (white bars) of 0.1 mM 8Br-cAMP. Results are shown as fold activation over control. Values are the mean of seven individual experiments performed in triplicate (±SEM). (**B**) MEHP targets the proximal *Star* promoter. A series of 5′ progressive deletions of the mouse *Star* promoter were transfected in MA-10 Leydig cells and treated with either dimethylsulfoxide (DMSO, white bars) or 150 µM MEHP (gray bars) in the presence (hatched and black bars) or absence (white and gray bars) of 0.1 mM 8Br-cAMP. Results are shown as fold activation over control. Values are the mean of four individual experiments performed in triplicate (±SEM). (**C**) MA-10 Leydig cells were treated with either dimethylsulfoxide (DMSO) or 100 µM MEHP with or without 0.1 mM 8Br-cAMP and whole cell extracts were prepared for immunodetection of C/EBPβ and SF1. αTUBULIN was used as a loading control. All experiments were repeated three times and produced similar results. *: *p* ≤ 0.05.

MEHP has been reported to decrease *Star* mRNA and protein levels in MA-10 Leydig cells and in primary Leydig cell cultures [41,51,52,54,55]. However, it is not known whether this is caused by a decrease in *Star* transcription. To test this possibility, a −980 bp mouse *Star* promoter fused to luciferase was transiently transfected in MA-10 Leydig cells which were exposed to increasing doses of MEHP (0.5, 10, 30, 75, and 150 µM). As shown in Figure 1A, MEHP had no effect on basal *Star* promoter activity. Treatment with 8Br-cAMP led to a 13-fold stimulation of the *Star* promoter as expected (Figure 1A). Co-treatment with 0.5 and 10 µM MEHP did not impact cAMP-mediated stimulation. However, higher MEHP doses (30, 75, and 150 µM) reduced cAMP-induced *Star* promoter activity by about 50% (Figure 1A).

To locate the MEHP-responsive region, a series of 5′ progressive deletion constructs of the mouse *Star* promoter were transfected in MA-10 Leydig cells treated with DMSO (vehicle) or 150 μM of MEHP. As shown in Figure 1B, the cAMP response was still decreased by MEHP up to −95 bp. The cAMP induction of a −70 bp *Star* reporter was no longer repressed by MEHP (Figure 1B). Thus the MEHP-responsive region seems to be located within the proximal *Star* promoter, a region that contains binding sites for several important transcription factors including SF1, NUR77, C/EBPβ, GATA4, and AP1 (reviewed in [56]). We therefore assessed whether MEHP decreased the levels of some of these transcription factors. As shown in Figure 1C, protein levels of both SF1 and C/EBPβ were not affected by MEHP.

**Figure 2:**
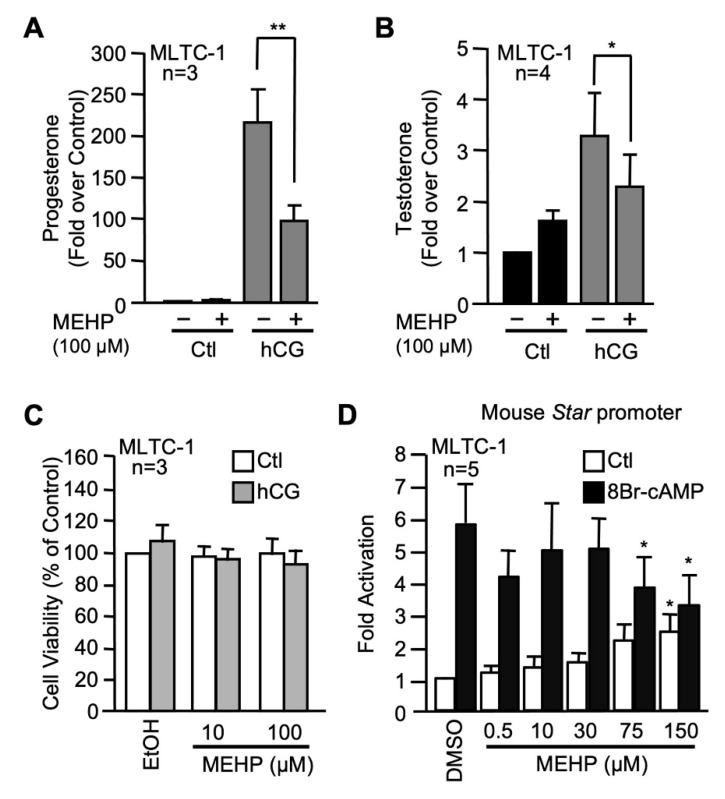
MEHP represses steroidogenesis in MLTC-1 Leydig cells. Progesterone (**A**) and testosterone (**B**) production were measured from MLTC-1 Leydig cells exposed to EtOH (−) or 100 µM MEHP (+) in the presence (gray bars) or absence (black bars) of 20 ng/mL of hCG. Values are the mean of 3 (progesterone) or 4 (testosterone) experiments each performed in duplicate (±SEM). (**C**) MEHP does not affect MLTC-1 Leydig cell viability. MLTC-1 Leydig cells were treated with either EtOH or two concentrations of MEHP (10, 100 µM) for 24 h in presence (gray bars) or absence (white bars) of 20 ng/mL of hCG. Cell viability was determined by performing MTT assays. Values are the mean of three individual experiments each performed in triplicate (±SEM). (**D**) MEHP represses cAMP-induced *Star* promoter activity in MLTC-1 Leydig cells. MLTC-1 cells were transfected with a −980 bp mouse *Star* reporter and treated for 24 h with either dimethylsulfoxide (DMSO) or increasing concentrations of MEHP in the presence (black bars) or absence (white bars) of 0.1 mM 8Br-cAMP. Results are shown as fold activation over control. Values are the mean of five individual experiments performed in triplicate (±SEM). *: *p* ≤ 0.05, **: *p* ≤ 0.01.

We next performed similar experiments in MLTC-1 Leydig cells to confirm that the effects of MEHP were not unique to the MA-10 Leydig cell line. Another advantage of the MLTC-1 cells is that they produce testosterone. We therefore tested the effect of 100 µM MEHP on progesterone (Figure 2A) and testosterone (Figure 2B) production by MLTC-1 Leydig cells. MEHP did not affect basal progesterone or testosterone production in MLTC-1 cells (Figure 2). Treatment with hCG led to an increase in steroid hormone production which was significantly reduced in the presence of MEHP (Figure 2A,B). MTT assay revealed that this decrease in the presence of MEHP was not caused by a reduction in cell viability or mitochondrial integrity (Figure 2C). Finally, MEHP also repressed cAMP-induced *Star* promoter activity in MLTC-1 cells (Figure 2D). A small but significant increase in *Star* basal promoter activity was observed at 150 µM MEHP. Thus, MEHP represses steroidogenesis by affecting cAMP/hormone-induced *Star* transcription in two Leydig cell lines.

### 2.2. MEHP Affects FDXR Expression

**Figure 3:**
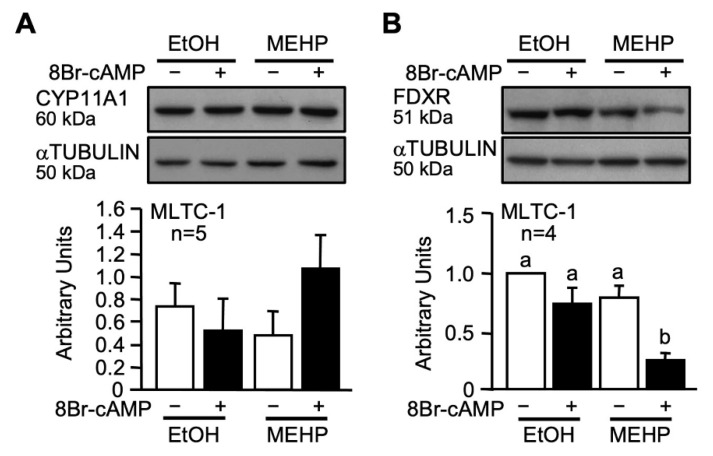
MEHP affects FDXR protein levels. MLTC-1 cells were treated for 24 h with either EtOH or 100 µM MEHP in the absence or presence of 0.1 mM 8Br-cAMP. Whole cell extracts were prepared and used for immunodetection of CYP11A1 (**A**) and FDXR (**B**). αTUBULIN was used as a loading control. All experiments were repeated four or five times as indicated and produced similar results. Values are the mean of densitometry of all individual experiments (±SEM). A different letter indicates a statistically significant difference (*p* ≤ 0.05).

Since hormone-induced progesterone production was affected in the presence of MEHP, we tested whether MEHP could also affect the expression of proteins involved in cholesterol conversion into pregnenolone. Expression of two key proteins, CYP11A1 (P450 side-chain cleavage) and FDXR (ferredoxin reductase), was analyzed by Western blotting. While CYP11A1 levels were unaffected (Figure 3A), FDXR protein levels were decreased in the presence of MEHP (Figure 3B). Thus, MEHP reduces the levels FDXR, a protein involved in cholesterol conversion, in addition to STAR, which is involved in cholesterol transport in the mitochondria.

### 2.3. Effects of Novel Green Plasticizers on Leydig Cell Function

**Figure 4:**
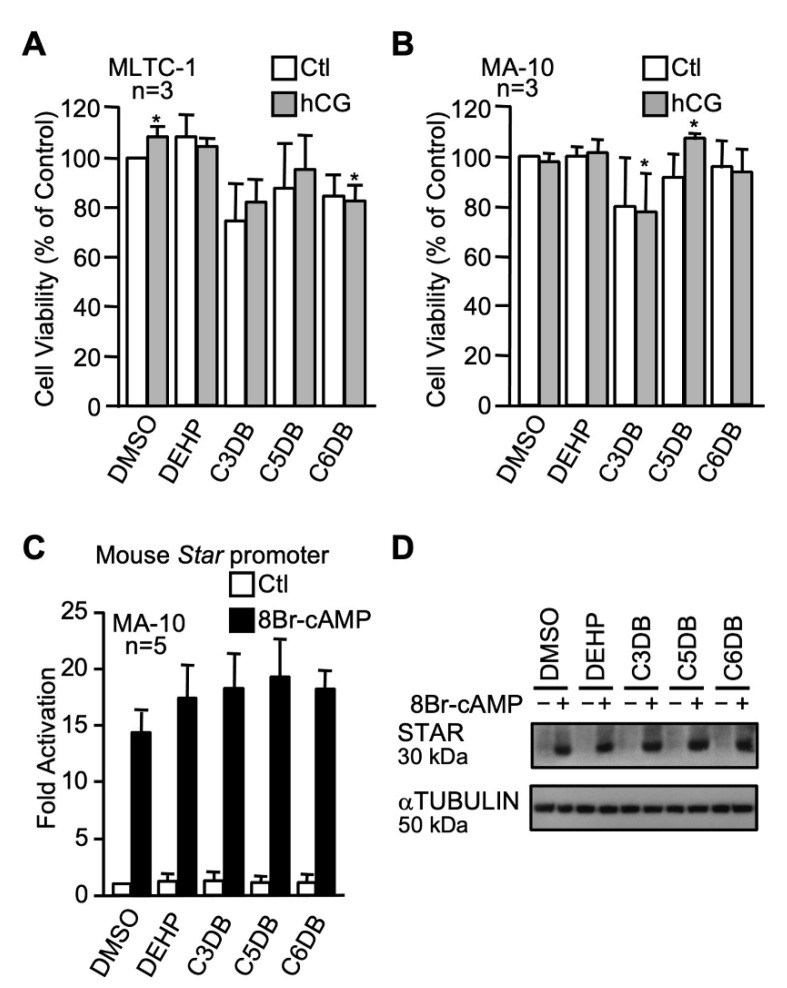
Effects of n-alkyl dibenzoate plasticizers on MA-10 and MLTC-1 Leydig cells. MA-10 (**A**) and MLTC-1 (**B**) cells were treated for 24 h with either dimethylsulfoxide (DMSO) or 100 µM of n-alkyl dibenzoate plasticizers as indicated in the presence (gray bars) or absence (white bars) of 20 ng/mL hCG and cell viability was determined by MTT assays. Values are the mean of three individual experiments each performed in triplicate (±SEM). (**C**) MA-10 Leydig cells were transfected with a −980 bp mouse *Star* reporter and treated with 100 µM of the different n-alkyl dibenzoate plasticizers as indicated in the absence (white bars) or presence (black bars) of 0.1 mM 8Br-cAMP. Results are shown as fold activation over control. Values are the mean of five individual experiments performed in triplicate (±SEM). (**D**) STAR protein levels were determined in MA-10 Leydig cells treated with vehicle (DMSO) or the various n-alkyl dibenzoate plasticizers as indicated in the absence (−) or presence (+) of 8Br-cAMP. αTUBULIN was used as a loading control. All experiments were repeated three times and produced similar results. *: *p* ≤ 0.05.

**Figure 5:**
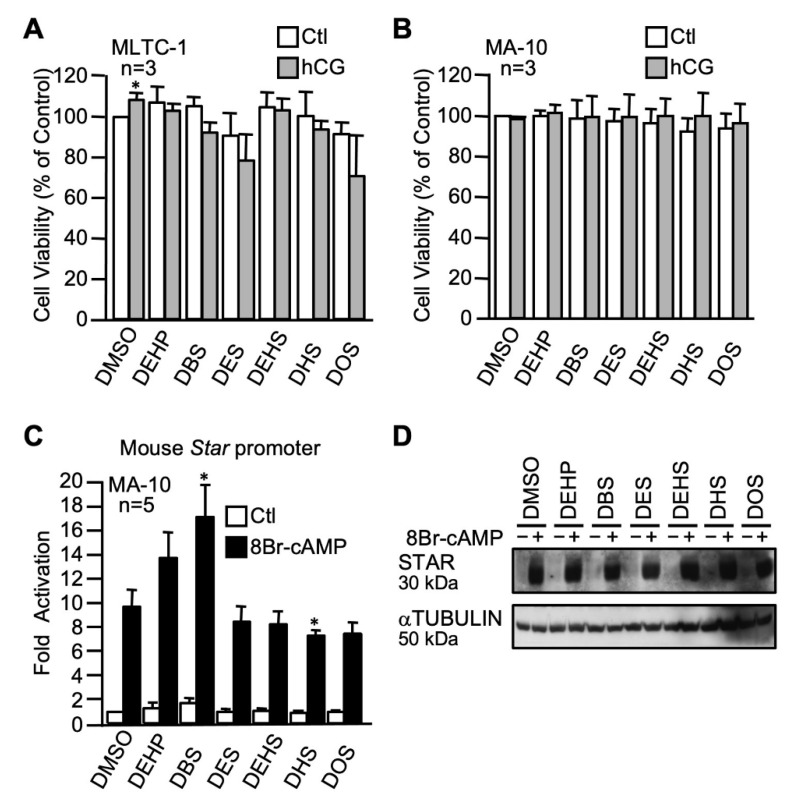
Effects of n-alkyl succinate plasticizers on MA-10 and MLTC-1 Leydig cells. MA-10 (**A**) and MLTC-1 (**B**) cells were treated for 24 h with either dimethylsulfoxide (DMSO) or 100 µM of n-alkyl succinate plasticizers as indicated in the presence (gray bars) or absence (white bars) of 0.1 mM hCG and cell viability was determined by MTT assays. Values are the mean of three individual experiments each performed in triplicate (±SEM). (**C**) MA-10 Leydig cells were transfected with a −980 bp mouse *Star* reporter and treated with 100 µM of the different n-alkyl succinate plasticizers as indicated in the absence (white bars) or presence (black bars) of 0.1 mM 8Br-cAMP. Results are shown as fold activation over control. Values are the mean of five individual experiments performed in triplicate (±SEM). (**D**) STAR protein levels were determined in MA-10 Leydig cells treated with vehicle (DMSO) or the various n-alkyl succinate plasticizers as indicated in the absence (−) or presence (+) of 8Br-cAMP. αTUBULIN was used as a loading control. All experiments were repeated three times and produced similar results. *: *p* ≤ 0.05.

**Figure 6:**
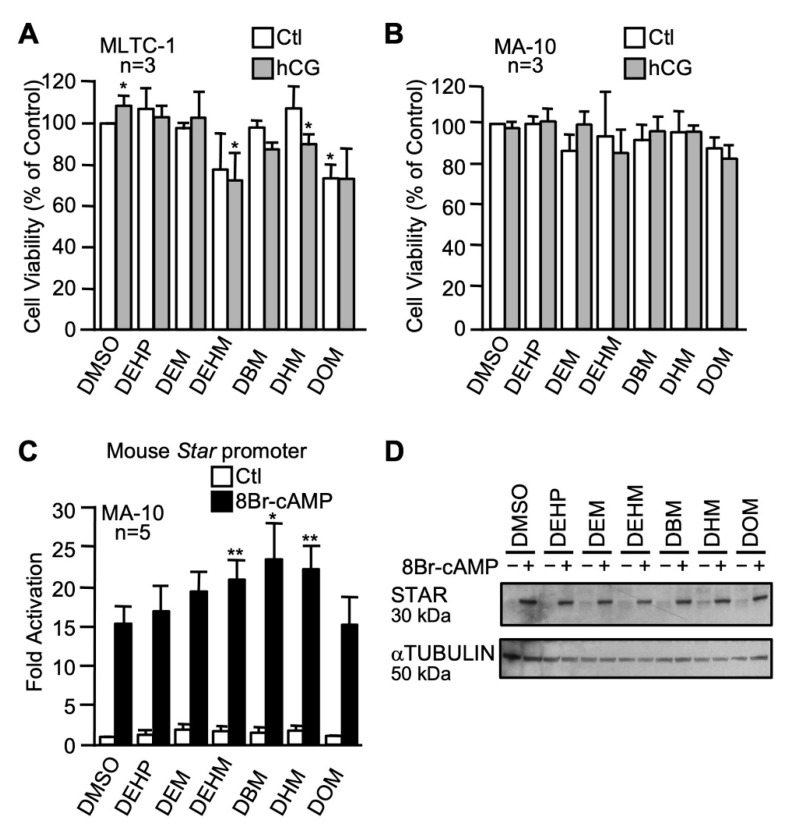
Effects of n-alkyl maleate plasticizers on MA-10 and MLTC-1 Leydig cells. MA-10 (**A**) and MLTC-1 (**B**) cells were treated for 24 h with either dimethylsulfoxide (DMSO) or 100 µM of n-alkyl maleate plasticizers as indicated in the presence (gray bars) or absence (white bars) of 20 ng/mL hCG and cell viability was determined by MTT assays. Values are the mean of three individual experiments each performed in triplicate (±SEM). (**C**) MA-10 Leydig cells were transfected with a −980 bp mouse *Star* reporter and treated with 100 µM of the different n-alkyl maleate plasticizers as indicated in the absence (white bars) or presence (black bars) of 0.1 mM 8Br-cAMP. Results are shown as fold activation over control. Values are the mean of five individual experiments performed in triplicate (±SEM). (**D**) STAR protein levels were determined in MA-10 Leydig cells treated with vehicle (DMSO) or the various n-alkyl maleate plasticizers as indicated in the absence (−) or presence (+) of 8Br-cAMP. αTUBULIN was used as a loading control. All experiments were repeated three times and produced similar results. *: *p* ≤ 0.05, **: *p* ≤ 0.01 when compared to control (DMSO + cAMP).

**Figure 7:**
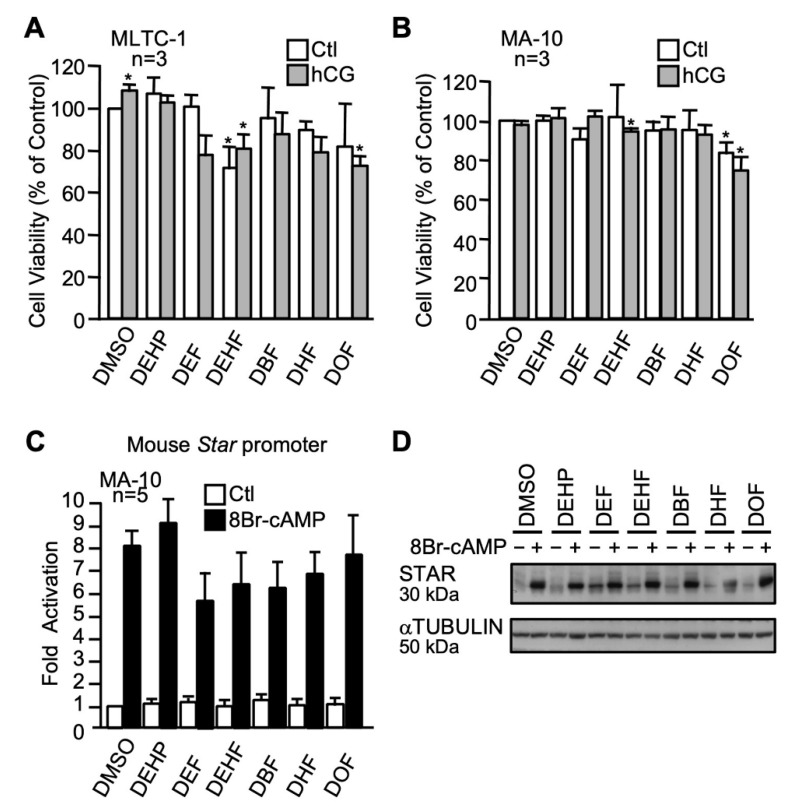
Effects of n-alkyl fumarate plasticizers on MA-10 and MLTC-1 Leydig cells. MA-10 (**A**) and MLTC-1 (**B**) cells were treated for 24 h with either dimethylsulfoxide (DMSO) or 100 µM of n-alkyl fumarate plasticizers as indicated in the presence (gray bars) or absence (white bars) of 20 ng/mL hCG and cell viability was determined by MTT assays. Values are the mean of three individual experiments, each performed in triplicate (±SEM). (**C**) MA-10 Leydig cells were transfected with a −980 bp mouse *Star* reporter and treated with 100 µM of the different n-alkyl fumarate plasticizers as indicated in the absence (white bars) or presence (black bars) of 0.1 mM 8Br-cAMP. Results are shown as fold activation over control. Values are the mean of five individual experiments performed in triplicate (±SEM). (**D**) STAR protein levels were determined in MA-10 Leydig cells treated with vehicle (DMSO) or the various n-alkyl fumarate plasticizers as indicated in the absence (−) or presence (+) of 8Br-cAMP. αTUBULIN was used as a loading control. All experiments were repeated three times and produced similar results. *: *p* ≤ 0.05.

**Figure 8:**
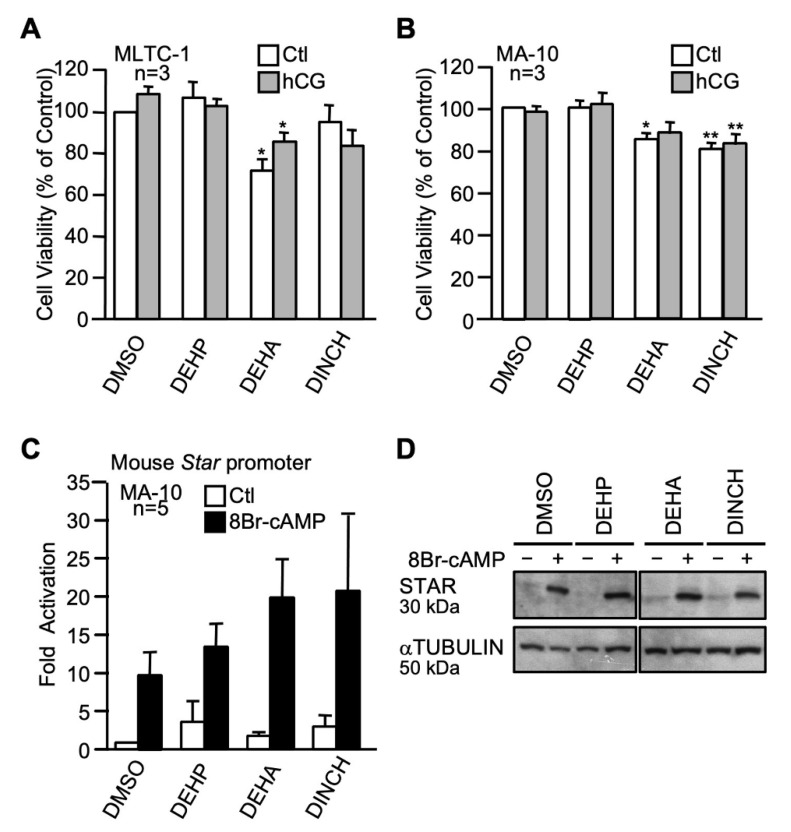
Effects of two commercial plasticizers on MA-10 and MLTC-1 Leydig cells. MLTC-1 (**A**) and MA-10 (**B**) Leydig cells were treated for 24 h with either DMSO or 100 µM of two commercial plasticizers (DEHA and DINCH) in presence (gray bars) or absence (white bars) of 20 ng/mL of hCG and assayed for cell viability using a MTT assay. Values are the mean of three individual experiments each performed in triplicate (±SEM). (**C**) MA-10 Leydig cells were transfected with a −980 bp mouse *Star* reporter and treated with 100 µM of the two commercial plasticizers as indicated in the absence (white bars) or presence (black bars) of 0.1 mM 8Br-cAMP. Results are shown as fold activation over control. Values are the mean of five individual experiments performed in triplicate (±SEM). (**D**) STAR protein levels were determined in MA-10 Leydig cells treated with vehicle (DMSO) or the two commercial plasticizers as indicated in the absence (−) or presence (+) of 8Br-cAMP. αTUBULIN was used as a loading control. All experiments were repeated three times and produced similar results. *: *p* ≤ 0.05, **: *p* ≤ 0.01.

Since phthalates are detrimental to Leydig cell gene expression and function, we tested the effects of novel biodegradable molecules that also have desirable plasticizing properties [57,58,59,60] on Leydig cell steroidogenesis as potential alternatives to phthalates. Four classes of novel plasticizers were tested: n-alkyl dibenzoate (Figure 4), n-alkyl diester succinate (Figure 5), n-alkyl diester maleate (Figure 6), and n-alkyl diester fumarate (Figure 7). Comparisons were also performed with two commercially available plasticizing molecules: Hexamoll-DINCH and diethylhexyl adipate (DEHA) (Figure 8).

#### 2.3.1. The n-alkyl Dibenzoate Plasticizer Series

Three plasticizers with different carbon chain lengths were used and compared to DEHP: 1,3-propanediol dibenzoate (C3DB), 1,5-pentanediol dibenzoate (C5DB), and 1,6-hexanediol dibenzoate (C6DB). Overall, cell viability of both MLTC-1 (Figure 4A) and MA-10 (Figure 4B) Leydig cell viability was not dramatically impaired by any of the n-alkyl dibenzoate plasticizers at 100 µM, although some small increases or decreases were noted. Luciferase gene reporter assays with the mouse *Star* promoter showed that all the n-alkyl dibenzoate plasticizers tested did not affect either basal or cAMP-induced *Star* promoter activity in MA-10 Leydig cells (Figure 4C). Consistent with this, all the n-alkyl dibenzoate plasticizers tested did not affect basal and cAMP-induced STAR protein levels in MA-10 Leydig cells (Figure 4D).

#### 2.3.2. The n-alkyl Succinate Plasticizer Series

For this plasticizer series, five different succinate derivatives were used at 100 µM and compared to 100 µM DEHP: dibutyl succinate (DBS), diethyl succinate (DES), diethylhexyl succinate (DEHS), dihexyl succinate (DHS), and dioctyl succinate (DOS). Although there was a tendency of reduced cell viability for some plasticizers (DEHS and DOS in MLTC-1), these effects did not reach statistical significance. Therefore, none of the five n-alkyl succinate plasticizers affected cell viability of both MLTC-1 (Figure 5A) and MA-10 (Figure 5B) Leydig cells in the presence of hCG or vehicle. The activity of the mouse *Star* promoter, either basal or cAMP-induced, was not affected by most of the n-alkyl succinate plasticizers, as revealed by transient transfection assays in MA-10 Leydig cells (Figure 5C). A statistically significant increase in cAMP-responsiveness was observed with DBS (17-fold compared to 9-fold for the control) while DHS significantly repressed the cAMP-induced stimulation (7-fold compared to 9-fold for the control). Basal and cAMP-induced STAR protein levels, however, were not affected by any of the five n-alkyl succinate plasticizers tested (Figure 5D).

#### 2.3.3. The n-alkyl Maleate Plasticizer Series

Five maleate plasticizers with different carbon chain lengths were used at a concentration of 100 µM and compared to DEHP: dibutyl maleate (DBM), diethyl maleate (DEM), diethylhexyl maleate (DEHM), dihexyl maleate (DHM), and dioctyl maleate (DOM). As shown in Figure 6A, a significant decrease in cell viability was observed in MLTC-1 Leydig cells after exposure to diethylhexyl maleate (DEHM) and dihexyl maleate (DHM) following hCG stimulation and with dioctyl maleate (DOM) in unstimulated cells only. However, no significant effect on the viability of MA-10 Leydig cells was observed with any of the n-alkyl maleate plasticizers (Figure 6B). In luciferase reporter assays, none of the n-alkyl maleate plasticizers repressed basal or cAMP-induced *Star* promoter activity in MA-10 Leydig cells (Figure 6C), while a slight but statistically significant increase in cAMP responsiveness was observed with diethylhexyl maleate (DEHM), dibutyl maleate (BDM), and dihexyl maleate (DHM). Basal and cAMP-induced STAR protein levels remained unchanged after exposure to the maleate plasticizers in MA-10 Leydig cells (Figure 6D).

#### 2.3.4. The n-alkyl Fumarate Plasticizer Series

Five fumarate plasticizers that differ by the length of their carbon chain were used at a concentration of 100 µM and compared to DEHP: dibutyl fumarate (DBF), diethyl fumarate (DEF), diethylhexyl fumarate (DEHF), dihexyl fumarate (DHF), and dioctyl fumarate (DOF). The viability of MLTC-1 Leydig cells was significantly impaired by DEHF (basal and hCG-stimulated) and DOF (in the presence of hCG) while the other fumarate plasticizers did not reduce cell viability (Figure 7A). MA-10 Leydig cell viability was only affected by DOF (basal and in the presence of hCG) and DEHF (in the presence of hCG) (Figure 7B). As revealed by luciferase reporter assays, none of the fumarate plasticizers affected basal and cAMP-induced *Star* promoter activity (Figure 7C). Western blots revealed that the n-alkyl fumarate plasticizers affected basal or cAMP-induced STAR protein levels to a different extent in MA-10 Leydig cells (Figure 7D). DBF, DEF, DEHF, and DHF increased basal STAR protein levels (Figure 7D). Exposure to DHF led to a decrease in cAMP-induced STAR protein levels (Figure 7D).

#### 2.3.5. The Commercial Plasticizers Hexamoll-DINCH and DEHA

We next evaluated the impact of 100 µM of two commercial plasticizers, Hexamoll-DINCH and diethylhexyl adipate (DEHA), on Leydig cells. When compared with the control (DMSO), DEHA caused a decrease in cell viability in unstimulated and hCG-stimulated MLTC-1 cells (Figure 8A) while both Hexamoll-DINCH and DEHA caused a decrease in cell viability in unstimulated and hCG-stimulated MA-10 cells (Figure 8B). Luciferase reporter assays in MA-10 Leydig cells showed that DEHA and Hexamoll-DINCH do not significantly affect basal or cAMP-induced *Star* promoter activity (Figure 8C). STAR protein levels were not affected by the two commercial plasticizers (Figure 8D).

### 2.4. Effect of Two Novel Candidate Plasticizers on Leydig Cell Line Function and FDXR Levels

**Figure 9:**
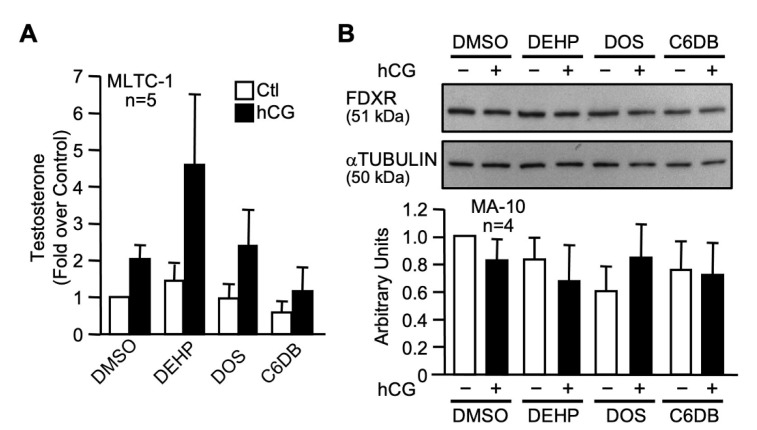
Effects of two green plasticizer candidates on Leydig cells steroidogenesis. (**A**) MLTC-1 Leydig cells treated with dimethylsulfoxide (DMSO) or 100 µM of DEHP, DOS, or C6DB in the absence (white bars) or presence (black bars) of 20 ng/mL hCG and testosterone levels in the media were determined by ELISA. Values are the mean of five individual experiments each performed in duplicate (±SEM). (**B**) MA-10 Leydig cells were treated with dimethylsulfoxide (DMSO) or 100 µM of DEHP, DOS, or C6DB in the absence (−) or presence (+) of 20 ng/mL hCG and whole cell extracts were prepared for immunodetection of the FDXR protein. αTUBULIN was used as a loading control. All experiments were repeated four times and produced similar results. Values are the mean of densitometry of the four individual experiments (±SEM).

We next assessed the impact of the C6DB and DOS candidate plasticizers (100 µM) on testosterone production by MLTC-1 Leydig cells. As shown in Figure 9A, exposure to DOS and C6DB did not affect testosterone production when compared to the control (DMSO). Finally, the effect of C6DB and DOS on FDXR protein levels, identified as a new target of MEHP, was assessed. As shown in Figure 9B, C6DB and DOS have no effect on FDXR protein levels.

## 3. Discussion

This study was designed to provide a better understanding of the molecular mechanisms of MEHP action in the inhibition of Leydig cells steroidogenesis and to assess the effects of novel plasticizers as alternatives to DEHP.

### 3.1. MEHP Represses Leydig Cells Steroidogenesis by Decreasing Star Gene Transcription

Numerous studies performed in rodents or using whole testis and Leydig cell lines have all shown that MEHP (the active metabolite of DEHP) negatively affects steroidogenesis [20,35,39,40,41,42]. Most cell line studies on the effect of MEHP on steroidogenesis were performed in the MA-10 Leydig cell line. Although a suitable model for studying the early steps of steroidogenesis, the MA-10 cell line produces mainly progesterone due to low and/or poorly active CYP17A1 [61], which makes it less suitable for studying the later steps of steroidogenesis. However, the MLTC-1 Leydig cell line has the ability to produce testosterone and constitutes an excellent model to characterize the impact of MEHP on the entire steroidogenic pathway. In our present study, exposure of both Leydig cell lines to 100 µM MEHP reduced hormone-induced testosterone and progesterone production by about 25 and 50%, respectively, without affecting the viability of the cells. We also observed a tendency towards increased basal steroidogenesis (without hCG stimulation), which is in agreement with other reports describing a stimulatory action of MEHP on basal steroidogenesis [62,63]. Interestingly, exposure to MEHP significantly decreased progesterone production more than testosterone, which indicates that MEHP may target the early steps of steroidogenesis more efficiently.

It is well established that MEHP represses Leydig cell steroidogenesis by decreasing STAR mRNA and protein levels, leading to a reduction in testosterone production [40,41,42,51,52]. Consistent with this, exposure of Leydig cells to MEHP was found to cause an increase in the number of lipid droplets, especially in hCG-treated cells ([64] and our unpublished data). Such an increase in lipid droplet content is consistent with a reduction in the activity of the cholesterol transport machinery, which includes the STAR protein. The mechanisms by which exposure to MEHP leads to reduced *Star* expression remain poorly characterized. In our present work, we found that exposure to MEHP significantly repressed cAMP-induced *Star* promoter activity while having no effect on basal *Star* promoter activity. This indicates that the MEHP-mediated reduction in STAR mRNA and protein levels is due, at least in part, to a decrease in hormone responsiveness of the *Star* promoter. An effect of MEHP on *Star* mRNA stability, however, cannot be excluded and more experiments are required to explore this possibility. Our functional promoter assays revealed that the MEHP-responsive region is located within the proximal region (-95 bp) of the *Star* promoter, a region that contains binding sites for several transcription factors important for *Star* expression including SF1, NUR77, C/EBPβ, GATA4, AP1 (cJUN/cFOS), and COUP-TFII [44,65,66,67,68,69,70,71,72,73]. When the protein levels of two transcription factors, SF1 and C/EBPβ, were assessed, no effects of MEHP were observed. Interestingly, another phthalate, MBP, was found to significantly reduce SF1 protein levels in MLTC-1 cells, although much higher concentrations (400, 800 µM) of MBP were used [74]. Our data on the action of MEHP on the *Star* promoter indicate that MEHP either targets other transcription factors known to act within this promoter region, affects post-translational modifications such as phosphorylation of any of these factors, or modulates their DNA-binding activity without affecting protein levels. Similar mechanisms were proposed for MBP, which was found to reduce GATA4 phosphorylation levels [74]. Further studies are needed to test these possibilities for MEHP action. Altogether, these data suggest that different phthalates might have different mechanisms of action to repress steroidogenesis in Leydig cells.

### 3.2. MEHP Reduces FDXR Protein Levels in Leydig Cells

Once cholesterol has reached the inner mitochondrial membrane, the next step is the conversion of cholesterol into pregnenolone. This reaction requires the action of two key proteins: CYP11A1 and FDXR. CYP11A1 is a mitochondrial enzyme that catalyzes the conversion of cholesterol into pregnenolone (reviewed in [50,75,76]). Ferredoxin reductase (FDXR, also known as adrenodoxin reductase, ADXR) is a mitochondrial FAD flavoprotein involved in the conversion of NADPH to NADP, a reaction that is essential for steroidogenesis since the electrons it produces are transferred to CYP11A1 and are required for its activity [76]. We found that CYP11A1 protein levels were unaffected by MEHP. On the other hand, FDXR protein levels dramatically decreased in the presence of MEHP to about 30% of control after cAMP treatment. Our identification of FDXR as a novel target of MEHP validates the hypothesis of Gunnarsson et al., who suggested that another site of MEHP action might exist prior to the formation of pregnenolone in the mitochondria [62].

Exposure of Leydig cells to MEHP is also known to cause a significant increase in the production of reactive oxygen species (ROS), further affecting Leydig cell function [52,55]. The exact mechanisms of increased ROS production remain to be elucidated. However, recent reports have revealed that reduced FDXR function in patients harboring hypomorphic mutations in the *FDXR* gene or in *Fdxr* mutant mice was associated with impairment of the electron transport chain, along with a significant increase in the production of ROS [77,78,79]. Our findings that exposure to MEHP reduces FDXR levels provide a mechanism to explain, at least in part, the MEHP-mediated increase in ROS production in Leydig cells.

### 3.3. Effects of Novel Candidate Plasticizers on Leydig Cell Steroidogenesis

Because of the detrimental effects of phthalates on male reproductive function, and the high cost associated with some of the currently used replacements, it became important to test novel candidate plasticizers. We tested the effects of four series of novel plasticizers (n-alkyl dibenzoate, n-alkyl diester succinate, n-alkyl diester maleate, n-alkyl diester fumarate) along with two commercially available plasticizers (Hexamoll-DINCH and DEHA) on *Star* promoter activity and STAR protein levels in Leydig cell lines.

None of the compounds in the dibenzoate series affected basal or cAMP-induced mouse *Star* promoter activity and STAR protein levels in MA-10 Leydig cells. This is in agreement with data from Boisvert et al., who reported that none of the dibenzoate plasticizers affected hormone-induced progesterone production in MA-10 Leydig cells [80]. However, a small decrease in cell viability was observed with 1,6 hexanediol dibenzoate (C6DB) and 1,3-propanediol dibenzoate (C3DB) in MLTC-1 and MA-10 Leydig cells, respectively. Despite the fact that C6DB caused slight toxicity in MLTC-1, it was chosen as a candidate green plasticizer because it is readily biodegraded by microorganisms and the monoesters produced are also quickly metabolized [81,82]. Furthermore, C6BD was not found to leach out of PVC compared to the other dibenzoate plasticizers and to DEHP [83].

For the succinate series, none were found to affect the viability of Leydig cells. In addition, most succinate plasticizers had no effect on *Star* promoter activity (basal or cAMP-induced) except for a small decrease observed in cAMP induction after treatment with dihexyl succinate (DHS). In agreement with our findings, succinate-derived plasticizers were also reported to have no effect on hCG-induced progesterone production [80]. Additionally, Kastner et al. reported that among that family of plasticizers, DOS has a very low ability to leach out from PVC [83]. The fact that DOS is not toxic to Leydig cells, does not affect steroidogenesis, and has a low potential to leach makes it an excellent replacement candidate.

For the maleate series, we found that several compounds negatively affected MLTC-1 Leydig cell viability. However, no inhibitory effects were observed on *Star* promoter activity or STAR protein levels following exposure to the maleate-derived plasticizers. On the other hand, Boisvert et al. reported that several of the maleate plasticizers caused a significant reduction in hormone-induced progesterone production in MA-10 Leydig cells [80]. These data, combined with our current data showing a lack of effects on *Star* expression, indicate that maleate-derived plasticizers affect steroidogenesis at a step after STAR action. Despite their desirable physicochemical properties [83], maleate plasticizers were found to be biodegraded as slowly as DEHP [58]. Based on these data, maleate plasticizers were eliminated from the list of potential plasticizers.

With respect to the fumarate series, several were toxic in both Leydig cell lines and negatively affected STAR protein levels. This is in agreement with the fact that several fumarate-derived plasticizers were found to significantly repress hormone-induced progesterone production in MA-10 cells [80]. In addition, they were reported to leach out of PVC after 3 weeks and displayed poor plasticizing properties [58]. These results indicate that fumarate plasticizers should be excluded from the list of potential phthalate alternatives.

The two commercial plasticizers tested, Hexamoll-DINCH and DEHA, were previously found not to leach out of PVC [83]. We found that both compounds decreased the viability of MA-10 and MLTC-1 Leydig cells. Similar results were obtained for DINCH by Boisvert et al. in MA-10 cells and they also reported that DINCH represses hormone-induced progesterone production in these cells [80]. Additional studies on these two compounds should be performed to fully assess their impact on male reproductive health.

Based on our present findings and data from the literature [80], two plasticizers (DOS and C6DB) were chosen for further examination. Basal and hormone-induced testosterone production, and evaluation of FDXR protein levels, revealed that DOS and C6DB did not significantly affect these Leydig cell parameters. This is consistent with two animal studies which reported that DOS had no significant systemic effects [84,85], suggesting that it could be a viable alternative plasticizer.

In conclusion, our data revealed that MEHP represses Leydig cell steroidogenesis by disrupting two of the earliest steps of steroidogenesis. The primary site of MEHP action seems to be the hormone-induced transport of cholesterol inside mitochondria by disrupting *Star* transcription, while another site of action includes the disruption of cholesterol conversion into pregnenolone by decreasing FDXR protein levels. Furthermore, two chemicals with desirable plasticizer properties, C6DB and DOS, were found to have no effect on Leydig cell steroidogenesis (STAR promoter activity, STAR and FDXR protein levels, testosterone production). Finally, our data also validated the MA-10 and MLTC-1 Leydig cell lines as two appropriate models for the study of MEHP action on steroidogenesis.

## 4. Materials and Methods

### 4.1. Chemicals

Di(2-ethylhexyl)phthalate (DEHP) and 8-bromo-cAMP (8Br-cAMP) were purchased from Sigma-Aldrich Canada (Oakville, Ontario, Canada). DEHP was dissolved in dimethyl sulfoxide (DMSO) purchased from Sigma-Aldrich Canada (Oakville, Ontario, Canada). MEHP (99% purity) was purchased from AccuStandard (New Haven, CT, USA) and dissolved in 99% ethanol. The new candidate plasticizers were provided by Dr. David G. Cooper (McGill University, Montréal, Canada) [57,58] and diluted in DMSO.

### 4.2. Cell Culture

Mouse tumor MA-10 Leydig cells [86] were provided by Dr. Mario Ascoli (University of Iowa, Iowa City, IA, USA) and cultured in Dulbecco’s Modified Eagle Medium-F12 (DMEM-F12) supplemented with penicillin and streptomycin and 15% horse serum and incubated at 37 °C in 5% CO_2_. Mouse tumor MLTC-1 Leydig cells were purchased from American Type Culture Collection (ATCC) and cultured in Dulbecco’s Modified Eagle Medium (DMEM) supplemented with penicillin and streptomycin and 10% fetal bovine serum (FBS) and incubated at 37 °C in 5% CO_2_. The phenotype of both MA-10 and MLTC-1 cell lines was ascertained by monitoring cell morphology and determining steroid hormone production. For all experiments, MA-10 and MLTC-1 were used between passages P10 and P25. For plasticizer treatments, cells were cultured in media containing charcoal-treated serum and treated with vehicle (DMSO or EtOH, none of which affects cell viability at the concentrations used) or 100 μM of plasticizer (DEHP/MEHP or new plasticizers). Twenty hours later, cells were stimulated with 0.1 mM 8Br-cAMP or hCG 20 ng/mL (LH analog) for 4 h.

### 4.3. Plasmids, Transfections and Luciferase Reporter Assays

The −980, −195, −144, −104, −95, −70 and −43 bp to +17 bp mouse *Star* promoter constructs were described previously [44,87]. MA-10 Leydig cells were seeded in 24-well plates at 150,000 cells per well and transfected the next day with 500 ng of reporter plasmid using JetPrime (PolyPlus-Transfections Inc., Illkirch, France) as described by the manufacturer. Cells were then lysed, and lysates analyzed as previously described [44,87]. These experiments were performed at least five times, each in triplicate.

### 4.4. Protein Purification and Western Blot

MA-10 Leydig cells were seeded in 6-well plates at 500,000 cells per well and treated as described above. Treated cells were rinsed twice with phosphate-buffered saline (PBS) and harvested for total protein extractions. Total proteins were isolated by lysing the cells with RIPA buffer [50 mM Tris-HCl (pH 7.5), 0.5% Igepal, 150 mM NaCl, 1 mM EDTA, 1 mM dithiothreitol (DTT), 0.5 mM phenylmethanesulfonyl fluoride (PMSF), 10 μg/mL aprotinin, 1 μg/mL leupeptin, and 1 μg/mL pepstatin] for 20 min at 4 °C, followed by one 10 s sonication pulse and centrifugation to remove cell debris. Protein concentrations were estimated using standard Bradford assay. Fifteen micrograms of total proteins were boiled for 2 min in a denaturing loading buffer [20% glycerol, 4% SDS, 100 mM Tris pH 6.8, 0.002% bromophenol blue, 4% β-mercaptoethanol], separated by SDS-PAGE, and transferred onto polyvinylidene difluoride (PVDF) membrane (Millipore, Bedford, MA, USA). For STAR and αTUBULIN, immunodetection was performed using an avidin-biotin approach according to the manufacturer’s instructions (Vector Laboratories, Inc., Ontario, Canada). For FDXR, CYP11A1, C/EBPβ and SF1 immunodetection, ECL-HRP detection kit (GE healthcare life sciences, Buckinghamshire, UK) was used according to the manufacturer’s instructions. Detection of STAR, FDXR, CYP11A1, C/EBPβ, SF1 and αTUBULIN was performed using an anti-STAR antiserum (FL-285, 1:1000 dilution, 200 ng/mL; Santa Cruz Biotechnologies, Santa Cruz, CA, USA), an anti-FDXR antiserum (sc-25846, 1:1000 dilution, 200 ng/mL; Santa Cruz Biotechnologies, Santa Cruz, CA, USA), an anti-CYP11A1 antiserum (sc-18043, 1:200 dilution, 200 ng/mL; Santa Cruz Biotechnologies, Santa Cruz, CA, USA), an anti-C/EBPβ antiserum (sc-150x, 1:5000 dilution, 400 ng/mL; Santa Cruz Biotechnologies, Santa Cruz, CA, USA), an anti-SF1 antiserum (1:1000 dilution, 1 µg/mL; ABR Affinity BioReagents, Golden, CO, USA) and a monoclonal anti-αTUBULIN antibody (1:10000 dilution, 600 ng/mL; Sigma-Aldrich Canada, Oakville, Ontario, Canada). αTUBULIN was used as a loading control. All experiments were repeated at least four times and produced similar results.

### 4.5. Progesterone and Testosterone Quantification

ELISA for progesterone and testosterone were performed using an EIA kit as recommended by the manufacturer (Cayman Chemical, Ann Arbor, MI, USA) as described in [88,89,90].

### 4.6. MTT Cell Assay

MA-10 and MLTC-1 Leydig cells were seeded in 96-well plates at 10,000 cells per well. The next day, cells (20,000 cells) were treated as described above in the cell culture section. The MTT cell assay measuring mitochondrial integrity and cell viability was performed as recommended by the manufacturer (Biotium, Hayward, Berkeley Heights, NJ, USA).

### 4.7. Statistical Analysis

For all single comparisons between two experimental groups, paired Student’s *t* tests were performed. For multiple group comparisons, statistical analyses were performed using one-way ANOVA followed by the Newman–Keuls post hoc test. For all statistical analyses, *p* ≤ 0.05 was considered significant. All statistical analyses were performed using GraphPad Prism software (La Jolla, CA, USA).

## Data Availability

All data generated or analyzed during this study are included in this article.
